# Transcriptomic Responses of Skeletal Muscle to Acute Exercise in Diabetic Goto-Kakizaki Rats

**DOI:** 10.3389/fphys.2019.00872

**Published:** 2019-07-09

**Authors:** Shuying Fu, Yuhuan Meng, Wenlu Zhang, Jiajian Wang, Yuting He, Lizhen Huang, Hongmei Chen, Jian Kuang, Hongli Du

**Affiliations:** ^1^School of Biology and Biological Engineering, South China University of Technology, Guangzhou, China; ^2^Department of Endocrinology, Guangdong General Hospital/Guangdong Academy of Medical Sciences, Guangzhou, China

**Keywords:** type 2 diabetes (T2D), skeletal muscle, acute exercise, Goto-Kakizaki (GK) rat, RNA-Seq

## Abstract

Physical activity exerts positive effects on glycemic control in type 2 diabetes (T2D), which is mediated in part by extensive metabolic and molecular remodeling of skeletal muscle in response to exercise, while many regulators of skeletal muscle remain unclear. In the present study, we investigated the effects of acute exercise on skeletal muscle transcriptomic responses in the Goto-Kakizaki (GK) rats which can spontaneously develop T2D. The transcriptomes of skeletal muscle from both 8-week-old GK and Wistar rats that underwent a single exercise session (60 min running using an animal treadmill at 15 m/min) or remained sedentary were analyzed by next-generation RNA sequencing. We identified 819 differentially expressed genes in the sedentary GK rats compared with those of the sedentary Wistar rats. After a single bout of running, we found 291 and 598 genes that were differentially expressed in the exercise GK and exercise Wistar rats when compared with the corresponding sedentary rats. By integrating our data and previous studies including RNA or protein expression patterns and transgenic experiments, the downregulated expression of *Fasn* and upregulated expression of *Tbc1d1*, *Hk2*, *Lpin1*, *Ppargc1a*, *Sorbs1*, and *Hmox1* might enhance glucose uptake or improve insulin sensitivity to ameliorate hyperglycemia in the exercise GK rats. Our results provide mechanistic insight into the beneficial effects of exercise on hyperglycemia and insulin action in skeletal muscle of diabetic GK rats.

## Introduction

Diabetes has become a growing global public health problem, leading to millions of deaths and considerable healthcare expenditure (Da et al., 2016). According to the International Diabetes Federation (IDF), 451 million people were estimated to have diabetes worldwide in 2017, with a projected increase to 693 million people by 2045 (Cho et al., 2018). Among these patients, 87–91% suffered from type 2 diabetes (T2D) which is characterized by hyperglycemia and insulin resistance in target organs (Chatterjee et al., 2017). In a short period, the prevalence of T2D in Asian populations has increased rapidly, with more than 60% of global T2D cases occurred in Asia. Moreover, it is increasingly recognized that Asian T2D patients may have different patterns from western counterparts, especially with a relatively lower mean body mass index (BMI) (Ramachandran et al., 2010; Yang and Weng, 2014). Therefore, suitable animal models are needed to study the pathophysiology and treatments of non-obese T2D patients.

The Goto-Kakizaki (GK) rat is one of the best spontaneous non-obese T2D animal models generated by repeated inbreeding of glucose intolerant Wistar rats, and has been extensively used in diabetic research (Portha et al., 2012; Hou et al., 2017). According to previous studies, GK rats had 10–20% lower body weights than control Wistar rats and exhibited hyperglycemia, hyperinsulinemia, and glucose intolerance at 8 weeks of age (Movassat et al., 2008; Almon et al., 2009). Moreover, insulin resistance and related signal transduction defects have also been reported in peripheral tissues such as skeletal muscle of adult GK rats (Nie et al., 2011). Due to these shared characteristics, GK rats may be a good model to study pathogenesis and treatments for T2D patients without obesity.

Physical activity, as a well-known beneficial factor, contributes to T2D both in prevention and treatment by improving glycemic control and insulin sensitivity (Praet and van Loon, 2008; Colberg et al., 2010; Borror et al., 2018). Skeletal muscle, one of the predominant sites of glucose disposal, plays a critical role in glycemic and metabolic homeostasis (DeFronzo and Tripathy, 2009). Benefits of physical activity are partly mediated by extensive metabolic and molecular remodeling of skeletal muscle responding to exercise (Egan and Zierath, 2013). It has been reported that the glucose uptake of skeletal muscle increased by up to 50-fold during bouts of exercise and remained elevated post exercise for several hours (Richter and Hargreaves, 2013; Sylow et al., 2017). Moreover, contraction-induced signal transduction can improve insulin resistant in skeletal muscle after exercise (Dela et al., 2018; Naufahu et al., 2018). Although considerable effort has been made to reveal comprehensive changes stimulated by exercise (Pilegaard et al., 2000; Mahoney et al., 2005; Catoire et al., 2012), many regulators of skeletal muscle have not yet been discovered, especially in the non-obese T2D.

To reveal the molecular responses of diabetic GK rats responding to a single bout of treadmill running, we assessed transcriptional profiles in skeletal muscle from both 8-week-old GK and Wistar rats with and without exercise by next-generation RNA sequencing (RNA-Seq) in the current study. The findings of molecular changes stimulated by exercise in skeletal muscle may provide the potential intervenient targets to treat and prevent deterioration in human T2D.

## Materials and Methods

### Animals

Male GK rats and age-matched male Wistar rats were purchased at the ages of 6 weeks from Shanghai SLAC Laboratory Animal Co., Ltd. (China) and were maintained under specific-pathogen-free (SPF) conditions with a temperature of 23–24°C, a 12:12-h light–dark cycle, and 50–60% atmospheric humidity. The animals received standard rat chow and water *ad libitum*. The study was approved by the institutional review board of the Guangdong Key Laboratory of Laboratory Animals. Research protocols conform to the guidelines of the Institutional Animal Care and Use Committee (IACUC) (Ethics Certificate No.: IACUC2014029).

### Exercise Training

All rats were familiarized with treadmill running using a progressive running program for 3 days at 7 weeks of age. On day 1, all rats ran at speeds of 8 m/min for 5 min, followed by 10 m/min for 10 min. On days 2 and 3, rats ran at speeds of 10 m/min for 5 min, followed by 15 m/min for 10 min. After the treadmill familiarization program, animals rested for 2 days. Then both GK and Wistar rats were randomly assigned to either a sedentary or an exercise group (Wistar, GK, exercise Wistar, exercise GK, *n* = 10/group). The formal acute exercise experiment was conducted at 8 weeks of age. The exercise groups (exercise Wistar, exercise GK) performed a single bout running at speeds 15 m/min for 60 min (Tsutsumi et al., 2015). Rats were encouraged to run by noise. During the exercise session, food and water were removed from both exercise and sedentary groups and immediately returned to cages after running.

### Tissue and Blood Sampling

Animals were anesthetized with pentobarbital sodium (50 mg/kg weight), and blood samples were collected from the abdominal aortic exsanguination using EDTA as an anticoagulant. Then gastrocnemius muscles were isolated, rapidly frozen in liquid nitrogen, and stored at -80°C. Plasma was centrifuged (2000 × *g*, 4°C, 15 min) from whole blood, and kept at -80°C before beginning the assays. The tissue and blood samples were collected at 2 h after acute exercise (Takimoto and Hamada, 2014; Pourteymour et al., 2017). Sedentary rats from both GK and Wistar groups were anesthetized at the same times coinciding with exercise groups.

### Assessment of Glucose and Insulin Levels in the Plasma

Blood glucose was measured by using FUJI DRI-CHEM SLIDE GLU-P III (FUJIFILM, Japan) with an automatic FUJI DRI-CHEM analyzer from plasma. Insulin was measured in plasma samples using commercial Rat Insulin ELISA Kits (Thermo Scientific, United States). Assays were conducted according to the manufacturer’s instructions. All standards and samples were assayed in triplicate.

### RNA Isolation, Library Preparation, and Sequencing

Totally, 24 gastrocnemius muscle samples (RNA from six animals in each subgroup) were prepared for RNA-Seq. Total RNA was isolated using TRIzol Reagent (Thermo Scientific, United States) according to the manufacturer’s instructions and resuspended in nuclease-free ultrapure dH_2_O. We used the Qubit^®^3.0 Fluorometer (Life Technologies, Pleasanton, CA, United States) to determine RNA purity. Then RNA integrity and concentration were assessed by the RNA Nano 6000 Assay Kit of the Bioanalyzer 2100 System (Agilent Technologies, Santa Clara, CA, United States). Sequencing libraries were prepared using NEBNext^®^Ultra^TM^ RNA Library Prep Kit for Illumina^®^(NEB, United States) and index codes were added to attribute sequences to each sample. Briefly, we used poly-T oligo-attached magnetic beads to purify mRNA samples from total RNA, and divalent cations under elevated temperature in NEBNext First Strand Synthesis Reaction Buffer (NEB, United States) to carry out fragmentation. The library fragments were purified with QIAquick PCR Kits (Qiagen, Germany) following first-strand and second-strand cDNA synthesized. A-tailing and adapter added were implemented to complete terminal repair, and we used agarose gel electrophoresis to retrieve the target products. After this step, PCR was performed to enrich templates and complete the library. The sequencing library quantitation was measured by the Qubit^TM^ 3.0 Fluorometer. Finally, the libraries were sequenced on an Illumina HiSeq X platform (Illumina, United States) to generate 150 bp paired-end reads after the clustering of the index-coded samples performed.

### Reads Filtering

Reads were discarded using the following filtering criteria to obtain clean reads: (1) reads with over 50% bases with quality less than 20; (2) reads with over 5% unidentified bases; and (3) reads with overrepresented adaptors.

### Differentially Expressed Genes

Clean reads from each sample were aligned to the *Rattus norvegicus* reference genome (*Rattus norvegicus*: Rnor 6.0 Ensembl version 86) using STAR2 (Dobin et al., 2013; Dobin and Gingeras, 2015) (version 020201). StringTie (Pertea et al., 2015) (version 1.3.0) was used to assemble the transcripts. StringTie also provides a Python script (prepDE.py) to extract read count information directly from the gtf generated by StringTie to build count matrices. The edgeR (Robinson and Smyth, 2008; Robinson and Oshlack, 2010; Lun et al., 2016), a bioconductor package, was used for differential expression analysis of read counts. Multiplicity correction was performed by applying the Benjamin–Hochberg method (Benjamini and Hochberg, 1995). Genes with false discovery rate (FDR) < 0.05 and absolute log2 fold change > 0.585 (1.5-fold) were considered as differentially expressed (Dickinson et al., 2018).

### Functional Enrichment Analysis

Differentially expressed genes (DEGs) were subjected to the Gene Ontology (GO), including biological process (BP), cell component (CC), and molecular function (MF), and the Kyoto Encyclopedia of Genes and Genomes (KEGG) pathway enrichment analysis using the clusterProfiler R package (Yu et al., 2012, 2015). The GO terms and KEGG pathways with a corrected *P*-value < 0.05 were considered to be significantly enriched.

### Protein–Protein Interaction (PPI) Network Analysis

The STRING (version 11.0) database was employed to construct the PPI network of DEGs with an interaction score >0.4 (Szklarczyk et al., 2015). Based on the interaction pair information, the PPI network was visualized in the Cytoscape software (version 3.5.1). The Molecular Complex Detection (MCODE) as a Cytoscape plugin was used to detect significant functional modules in the PPI network with default parameter settings: degree cutoff of 2, node score cutoff of 0.2, and K-core of 2 (Bader and Hogue, 2003).

### Transcriptional Regulatory Network Analysis

The Match tool in the TRANSFAC database was used to predict potential transcriptional factors (TFs) based on the 2000 bp upstream of transcription start sites of DEGs (Szklarczyk et al., 2015). The TFs with a relative score <0.9 were filtered out. A transcriptional regulatory network was constructed among the DEGs and their corresponding TFs and visualized by Cytoscape software.

### Quantitative Real-Time PCR (qRT-PCR) Validation

In order to validate the gene expression changes, total RNA was reverse transcribed using Primescript^TM^ RT Reagent Kit with gDNA Eraser (Takara, Japan), and qRT-PCR was performed with SYBR^®^Premix Ex Taq^TM^ II (Takara, Japan). Reactions were run in a Light Cycler 96 System (Roche, Switzerland). Relative expression ratios were calculated using beta-actin (*Actb*) as a housekeeping gene and comparative cycle threshold method (2^-ΔΔCt^). The primer sequences for these genes were designed by Primer Express 6.0, and the details were listed in Supplementary Table S1.

### Statistical Analysis

For metabolic measurements, the data are represented as means ± SEM. Two-way analysis of variance (ANOVA) with Tukey’s *post hoc* analysis was performed to identify differences among groups. Statistical analysis was carried out using GraphPad Prism (version 7), and a *P*-value < 0.05 was considered to be statistically significant.

### Data Availability

The RNA-Seq raw data have been submitted to the NCBI SRA database (PRJNA490045).

## Results

### Animal Characteristics

To determine the effects of acute exercise, plasma glucose and insulin levels were analyzed. For glucose levels, two-way ANOVA analysis revealed rat strain (GK > Wistar, *P* < 0.01) and exercise (sedentary > exercise, *P* < 0.05) had significant main effects. *Post hoc* analysis showed that plasma glucose levels of GK rats were higher than Wistar rats and reached the diagnostic criteria for T2D (the random glucose > 11.1 mmol/L) (*P* < 0.01) at the 8 weeks of age (Supplementary Figure S1A). At 2 h post exercise, the levels of plasma glucose were similar between the sedentary and exercise Wistar groups while glucose levels showed distinct decreases in the exercise GK group compared with sedentary one (*P* < 0.05) (Supplementary Figure S1A). Although a single bout of running relieved hyperglycemia in exercise GK rats, the glucose levels remained significantly higher than in Wistar rats (*P* < 0.01) (Supplementary Figure S1A). For plasma insulin, rat strain (GK > Wistar, *P* < 0.01) and exercise (sedentary > exercise, *P* < 0.01) also had significant main effects. The insulin levels were significantly elevated in the GK rats compared with the Wistar rats (*P* < 0.01) (Supplementary Figure S1B). After acute exercise, insulin did not differ between two Wistar groups, but the levels in exercise GK rats displayed significant decreases in comparison with sedentary GK rats (*P* < 0.01) (Supplementary Figure S1B).

### Identification of Differentially Expressed Genes

**Figure 1:**
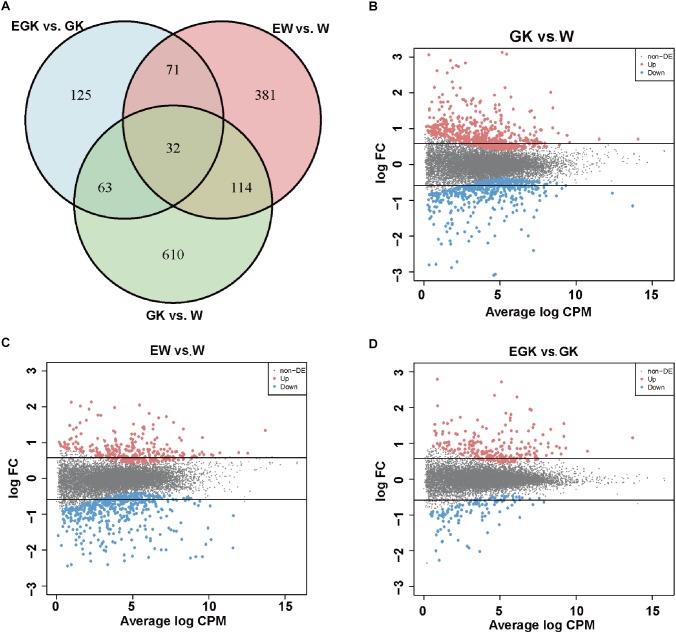
The number of DEGs. **(A)** Venn diagram representing the overlap of DEGs in skeletal muscle among the three comparisons (GK vs. W, EGK vs. GK, and EW vs. W). MA plots representing the distribution of DEGs in GK **(B)**, EW **(C)**, and EGK **(D)**. The dots between two horizontal lines indicate absolute fold change <1.5, red dots indicate significantly upregulated genes, and blue dots indicate significantly downregulated genes (FDR < 0.05). W, Wistar; EW, exercise Wistar; GK, Goto-Kakizaki; EGK, exercise GK.

To investigate the regulatory mechanisms involved in response to acute exercise, we analyzed the transcriptomes of skeletal muscle from four groups (Wistar, GK, exercise Wistar, and exercise GK). Among the 32,494 surveyed genes, 12,729 genes [kept genes with at least 1 count-per-million reads (CPM) in at least 12 samples] were considered as a quantifiable dataset. A total of 1396 DEGs were identified in three comparisons (GK vs. Wistar, exercise GK vs. GK, and exercise Wistar vs. Wistar) and the details of these genes were presented in Supplementary Table S2. There were 819 genes whose expression was significantly altered in GK rats relative to Wistar rats (Figure 1A). Of these DEGs identified in GK vs. Wistar comparison, 455 genes were upregulated, and 364 genes were downregulated (Figure 1A,B). A single session of running in Wistar rats resulted in 598 DEGs in skeletal muscle, with 215 upregulated and 383 downregulated (Figure 1A,C). However, exercise only induced 291 DEGs in GK rats, of which 194 genes were upregulated and 97 genes were downregulated (Figure 1A,D). Venn diagram analysis revealed that 32 DGEs were common in three comparisons, and 610, 381, and 125 DGEs were unique to GK vs. Wistar, exercise Wistar vs. Wistar, and exercise GK vs. GK, respectively (Figure 1A). These results indicated that transcriptomes of skeletal muscle varied widely between GK and Wistar rats. Moreover, the magnitude of transcriptional changes responding to exercise in the Wistar rats was greater than that in the GK rats. To validate RNA-Seq data, several DEGs were selected for validation of expression by qRT-PCR analysis (Supplementary Figure S2). The results exhibited similar expression patterns between RNA-Seq and qRT-PCR, and the correlation coefficient was 0.95 (*P* < 0.0001), which indicated the reliability of RNA-Seq data (Supplementary Figure S2).

### Functional Enrichment and PPI Analysis of Differentially Expressed Genes Between GK and Wistar Rats

**Figure 2:**
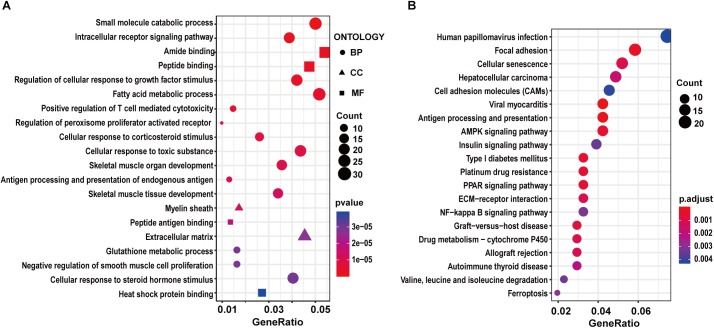
GO and KEGG enrichment analysis of DEGs in GK vs. Wistar comparison. The top 20 significantly enriched GO terms **(A)** and KEGG pathways **(B)**.

To characterize the potential functions altered by the DEGs, we carried out GO and KEGG pathway enrichment analysis. For the GK vs. Wistar comparison, we found DEGs were significantly enriched in the muscle-related GO terms such as skeletal muscle organ development, skeletal muscle tissue development, and negative regulation of smooth muscle cell proliferation among the top 20 terms (Supplementary Table S3 and Figure 2A). Moreover, some enriched GO terms related to metabolism were found, including small molecular catabolic process, intracellular receptor signaling pathway, fatty acid metabolic process, and glutathione metabolic process. In the KEGG pathway enrichment analysis, several significant pathways were related to diabetes, including AMPK signaling pathway, PPAR signaling pathway, insulin signaling pathway, and so on (Supplementary Table S3 and Figure 2B). These functional enrichment results suggested that GK rats might have significant changes in energy metabolism and signal transduction compared to Wistar rats, which might contribute to the development of T2D. Among these enriched pathways, five genes (*Slc27a1*, *Fasn*, *Tbc1d1*, *Pfkfb3*, and *Igf1r*) related to the regulation of glucose homeostasis were screened. Of these five genes, the expression of *Slc27a1* (solute carrier family 27, member 1), *Fasn* [fatty acid synthase (FASN)], and *Tbc1d1* (TBC1 domain family member 1) was significantly increased in the GK rats compared with Wistar rats. In contrast, the expression of *Pfkfb3* (6-phosphofructo-2-kinase) and *Igf1r* (insulin-like growth factor 1) was significantly decreased in the GK rats. To reveal functional interactions between DEGs, we constructed a PPI network by mapping genes via the STRING database and identified densely connected networks using the MCODE plugin in Cytoscape. Twenty-three models were obtained and the highest score module consisted of 14 nodes and 87 edges (Supplementary Figure S3A).

### Functional Enrichment and PPI Analysis of Differentially Expressed Genes Between Exercise Wistar and Wistar Rat

**Figure 3:**
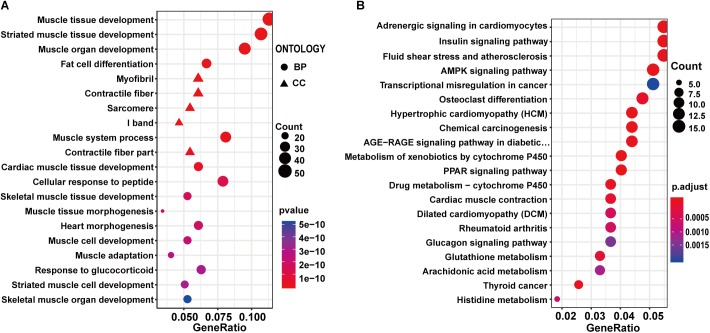
GO and KEGG enrichment analysis of DEGs in exercise Wistar vs. Wistar comparison. The top 20 significantly enriched GO terms **(A)** and KEGG pathways **(B)**.

To investigate the effect of exercise on healthy control rats, the 598 DEGs identified between exercise Wistar and Wistar rats were subjected to functional enrichment analysis. The top 20 significantly enriched GO terms were mainly associated with muscle, including muscle tissue development, striated muscle tissue development, muscle organ development, muscle system process, and so on (Supplementary Table S3 and Figure 3A). The GO enrichment analysis indicated that the development of muscle underwent significant changes in the exercise Wistar rats. The KEGG pathway analysis showed that insulin signaling pathway, AMPK signaling pathway, PPAR signaling pathway, and glucagon signaling pathway associated with metabolism were significantly enriched, indicating that exercise also affected metabolism in the skeletal muscle of Wistar rats (Supplementary Table S3 and Figure 3B). Moreover, we performed the PPI network analysis of DEGs and extracted eight significantly clustered modules. The module with the highest score had 24 gene nodes and 134 edges (Supplementary Figure S3B).

### Functional Enrichment and PPI Analysis of Differentially Expressed Genes Between Exercise GK and GK Rats

**Figure 4:**
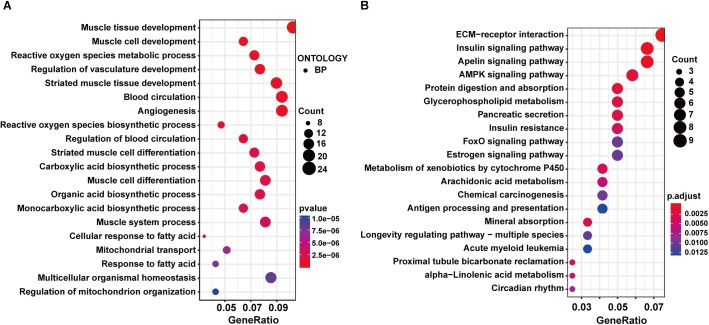
GO and KEGG enrichment analysis of DEGs in exercise GK vs. GK comparison. The top 20 significantly enriched GO terms **(A)** and KEGG pathways **(B)**.

The GO enrichment analysis in the exercise GK vs. GK comparison showed that DEGs were enriched in the GO terms related to muscle, including muscle tissue development, muscle cell development, and so on (Supplementary Table S3 and Figure 4A). These results were consistent with the other two comparisons. Moreover, carboxylic acid biosynthetic process, organic acid biosynthetic process, monocarboxylic acid biosynthetic process, and response to fatty acid were identified in the top 20 GO terms, indicating the metabolism might be significantly changed after exercise in the GK rats. In the top 20 enriched pathways, insulin signaling pathway, AMPK signaling pathway, glycerophospholipid metabolism, insulin resistance, and FoxO signaling pathway related to metabolism and diabetes were identified (Supplementary Table S3 and Figure 4B). We also carried out the PPI network analysis using the DEGs induced by exercise in the GK rats. Eight significant modules in the PPI network were identified, and the highest score module was visualized and presented in Supplementary Figure S3C, which contained 14 genes with 38 edges.

**Figure 5:**
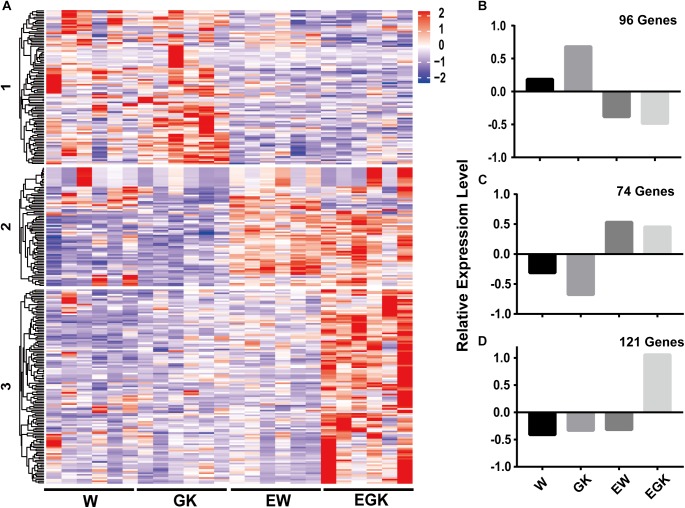
The expression patterns of DEGs of exercise GK in the four groups. DEGs in the exercise GK rats were analyzed using *k*-means clustering method. This identified three major groups, presented in heat map based on patterns of expression **(A)**, and graphical format based on the mean value of *z*-score values of gene expression in cluster 1 **(B)**, cluster 2 **(C)**, and cluster 3 **(D)**.

To gain a further understanding of how exercise exerts the effect on diabetic GK rats, we analyzed the expression patterns of 291 DEGs between exercise GK and GK rats using *k*-means clustering, and three major groups were clustered (Figure 5A and Supplementary Table S4). The first cluster revealed high average levels of expression in sedentary groups (Wistar and GK rats), decreasing after exercise (exercise Wistar and exercise GK) (Figure 5A,B). In contrast, the second cluster exhibited low average levels of expression in sedentary groups, increasing after exercise (Figure 5A,C). The DEGs in cluster 1 were significantly enriched in carbohydrate digestion and absorption, and fatty acid metabolism pathways (Supplementary Table S4). The expression of gene *Fasn* (FASN) was significantly downregulated in both Wistar and GK groups after exercise (Table 1). In cluster 2, pathways significantly enriched for DEGs were related to glycerophospholipid metabolism, AMPK signaling, and so on (Supplementary Table S4). In the glycerophospholipid metabolism pathway, the expression of gene *Lpin1* (Lipin-1) was significant upregulation in both exercise strains (Table 1). Moreover, the expression of *Tbc1d1* (TBC1 domain family member 1) involved in AMPK signaling pathway was also significantly upregulated in two exercise groups (Table 1).

**Table 1:** The details of key DEGs in the exercise GK rats and other datasets.

Cluster	Gene	FC (exercise/control)	FDR	References
1	*Fasn*	-1.52	2.68*E*-03	Current study
2	*Lpin1*	1.98	8.67*E*-07	Current study
2	*Lpin1*	1.32	2.88*E*-02	GSE28422 (Vissing and Schjerling, 2014)
2	*Tbc1d1*	1.79	6.26*E*-06	Current study
2	*Tbc1d1*	1.84	2.80*E*-03	GSE28422 (Vissing and Schjerling, 2014)
3	*Hk2*	1.65	7.01*E*-04	Current study
3	*Hk2*	1.55	1.97*E*-02	GSE59363 (Hansen et al., 2015)
3	*Ppargc1a*	3.11	3.33*E*-25	Current study
3	*Ppargc1a*	2.58	3.00*E*-04	GSE59363 (Hansen et al., 2015)
3	*Ppargc1a*	1.91	2.00*E*-04	GSE28422 (Vissing and Schjerling, 2014)
3	*Ppargc1a*	2.26	4.00*E*-03	GSE10533 (Allen et al., 2009)
3	*Sorbs1*	1.80	2.39*E*-06	Current study
3	*Sorbs1*	2.05	6.00*E*-04	GSE28422 (Vissing and Schjerling, 2014)
3	*Hmox1*	1.84	7.21*E*-06	Current study
3	*Hmox1*	1.83	1.27*E*-02	GSE59363 (Hansen et al., 2015)

*FC, fold change; FDR, false discovery rate*.

Interestingly, we found that the third cluster was distinctive, with high average levels of expression only in exercise GK rats (Figure 5A,D). These DEGs whose expression was dramatically upregulated after exercise in GK rats might provide some critical clues regarding pathways and genes to reveal the mechanisms of improving hyperglycemia. For cluster 3, a total of 29 significantly enriched pathways were identified, and several pathways were associated with diabetes, including insulin signaling pathway, insulin resistance, glycolysis/gluconeogenesis, FoxO signaling pathway, HIF-1 signaling pathway, and type 2 diabetes mellitus (Supplementary Table S4). Among these pathways, some key regulators *Hk2* [hexokinase 2 (HKII)], *Ppargc1a* (PPARG coactivator 1 alpha), *Sorbs1* (sorbin and SH3 domain containing 1), *Hmox1* [heme oxygenase 1 (HO-1)] which had been reported involving in regulation of glucose homeostasis, were significantly upregulated induced by exercise in GK rats (Table 1).

### Transcriptional Regulatory Network Analysis

**Figure 6:**
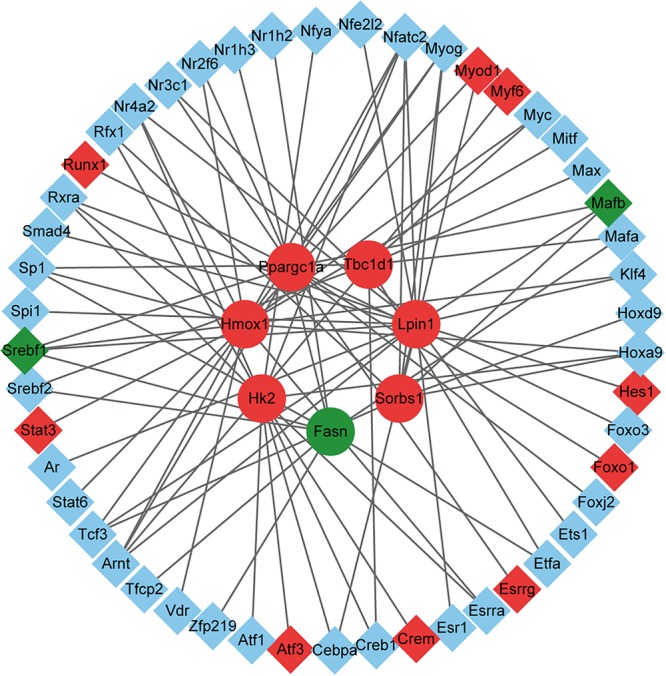
The network of DEGs and their corresponding TFs. The circles represent the target genes of the TFs, and the diamonds represent the TFs. Red indicates significantly upregulated, green indicates significantly downregulated, and blue indicates the expression of genes without significant difference.

Through the above analysis, we identified seven key DEGs in the exercise GK rats, including *Fasn*, *Tbc1d1*, *Lpin1*, *Hk2*, *Ppargc1a*, *Sorbs1*, and *Hmox1*. To explore factors affecting the expression of these DEGs, we used the TRANSFAC database to predict potential TFs. A total of 50 potential TFs were involved in the regulation of these key genes (Supplementary Table S5). The number of TFs that regulated the genes *Fasn*, *Tbc1d1*, *Lpin1*, *Hk2*, *Ppargc1a*, *Sorbs1*, and *Hmox1* were 19, 12, 18, 18, 12, 8, and 10, respectively. Of these predicted TFs, 11 were significantly altered in the exercise GK rats, with 2 downregulated and 9 upregulated (Figure 6). These differentially expressed TFs might lead to the altered expression of genes *Fasn*, *Tbc1d1*, *Lpin1*, *Hk2*, *Ppargc1a*, *Sorbs1*, and *Hmox1*, which might help for understanding the transcriptomic responses in the exercise GK rats.

## Discussion

Skeletal muscle plays an important role in maintaining systemic glucose homeostasis because it is one of the primary organs for glucose metabolism. Substantial evidence indicated that physical activity plays an essential role in the prevention and treatment of T2D and even a single bout of exercise can improve hyperglycemia and insulin resistance (Colberg et al., 2010; Richter and Hargreaves, 2013). However, the molecular mechanisms underlying the beneficial effects are still unclear, and few studies were forced on the non-obese T2D which often occurred in the Asian diabetic patients. The GK rat, a spontaneously non-obese T2D model, displayed hyperglycemia, insulin resistance, and glucose intolerance at the 8 weeks age (Movassat et al., 2008; Almon et al., 2009; Portha et al., 2012). Considering these characteristics, GK rats are a suitable model for studying the exercise effects on the non-obese T2D. In the current study, the transcriptomes of skeletal muscle from both 8-week-old GK and Wistar rats that underwent a single bout of exercise (60 min running using an animal treadmill at 15 m/min) or not were analyzed by RNA-Seq technology (Bedford et al., 1979; Tsutsumi et al., 2015). The differential expression analysis showed that 819 genes were significantly changed between GK rats and Wistar rats. At 2 h post exercise, 598 and 291 DEGs were identified in the exercise Wistar and exercise GK rats, respectively, compared with the corresponding sedentary rats. To reveal the acute exercise induced specific transcriptional adaptions involved in diabetic GK rats, 291 DEGs found in exercise GK rats were further clustered to three groups based on the expression patterns. There were three genes (*Fasn*, *Lpin1*, and *Tbc1d1*) found in clusters 1 and 2, which were significantly altered in both exercise Wistar and exercise GK rats. Moreover, four genes (*Hk2*, *Hmox1*, *Sorbs1*, and *Ppargc1a*) which were significantly upregulated in the exercise GK rats were identified in cluster 3. By integrating our data and previous studies including RNA or protein expression patterns and transgenic experiments of these key genes, we predicted that the seven key genes might be associated with the improvement of hyperglycemia or insulin sensitivity.

Among these four DEGs which were significantly upregulated in the exercise GK rats, the gene *Hk2* encodes hexokinase II which catalyzes glucose to glucose-6-phosphate, the first rate-limiting step in glycolysis. It has been reported that the expression of *Hk2* was reduced in skeletal muscle of diabetes (Braithwaite et al., 1995; Esteves et al., 2018). In the current study, the gene *Hk2* was significantly upregulated in the exercise GK rats, which was consistent with previous studies that the expression of *Hk2* and HKII protein content was significantly increased in the T2D or obese subjects after exercise (Croymans et al., 2013; Hansen et al., 2015). Moreover, transgenic studies showed that partial *Hk2* knockout in mice impaired glucose uptake and suppressed insulin action (Fueger et al., 2007), while overexpression of *Hk2* enhanced exercise-stimulated glucose uptake in high-fat-fed mice (Fueger et al., 2004). In light of these, the upregulated expression of *Hk2* in exercise GK rats might promote glycolysis, increase glucose uptake, and improve insulin sensitivity. The gene *Ppargc1a* encodes peroxisome proliferator-activated receptor gamma coactivator 1 alpha (PGC-1α) which is a transcription coactivator regulating energy metabolism. Accumulating evidence suggested that PGC1-α was associated with the regulation of T2D (Wu et al., 2016). In the diabetic muscle, the expression of *Ppargc1a* was significantly decreased, which might contribute to insulin resistance (Mootha et al., 2003; Patti et al., 2003; Chan and Arany, 2014). Interestingly, recent studies indicated that *Ppargc1*a was upregulated after exercise in skeletal muscle of T2D rodents and patients, improving glucose homeostasis and insulin sensitivity (Allen et al., 2009; Summermatter et al., 2013; Vissing and Schjerling, 2014; Hansen et al., 2015). In addition, transgenic increased expression of *Ppargc1a* in the skeletal muscle enhanced glucose uptake (Wende et al., 2007; Zhang et al., 2013; Wu et al., 2016). Therefore, increased expression in *Ppargc1a* might be associated with improvement of hyperglycemia in the exercise GK rats. Cbl-associated protein (CAP), encoding by *Sorbs1*, played a positive role in the insulin signaling pathway (Baumann et al., 2000). The genetic variation of *Sorbs1* was associated with the incidence of diabetes and glucose homeostasis, supporting the involvement of *Sorbs1* in the pathogenesis of diabetes (Chang et al., 2018). Besides, the expression of *Sorbs1* was correlated negatively with homeostatic model assessment for insulin resistance (HOMA-IR). In other words, overexpression of *Sorbs1* meant higher insulin sensitivity (Pettersson et al., 2013). The expression of *Sorbs*1 was increased after acute exercise, which might be related to the amelioration of glucose levels in the exercise GK rat (Vissing and Schjerling, 2014). The gene *Hmox1* encodes the enzyme HO-1 associated with insulin sensitivity and protecting cellular defense from oxidative stress (Ndisang et al., 2010). According to previous studies, the expression of *Hmox1* in skeletal muscle of T2D decreased significantly, but it increased markedly after exercise, which was consistent with our data (Bruce et al., 2003; Hansen et al., 2015). Besides, it has been demonstrated that the use of hemin to induce *Hmox1* improved glucose tolerance and enhanced insulin sensitivity in the GK rats (Ndisang and Jadhav, 2009). Collectively, these data suggested that the upregulated expression of *Hk2*, *Ppargc1a*, *Sorbs1*, and *Hmox1* might be implicated in the improvement of hyperglycemia and insulin resistance in the exercise GK rats.

Three genes (*Fasn*, *Lpin1*, and *Tbc1d1*) associated with insulin sensitivity and glucose homeostasis had significant changes in both exercise GK and Wistar rats. It has been known for many years that the dysregulated lipid metabolism leads to lipotoxicity which implicated in insulin resistance and T2D. FASN plays a vital role in lipogenesis via converting acetyl-CoA and malonyl-CoA to palmitate. Overexpression of *Fasn* has been associated with insulin resistance and diabetes (Menendez et al., 2009). In contrast, muscle-specific *Fasn* knockout ameliorated insulin resistance, glucose intolerance, and increased glucose uptake in high-fat diet fed mice (Funai et al., 2013). In the GK skeletal muscle, the significantly upregulated *Fasn* might be associated with insulin resistance to cause hyperglycemia and hyperinsulinemia. After a single bout of running, we found that *Fasn* expression was significantly decreased, which might improve insulin sensitivity in both two exercise groups and attenuate plasma glucose in GK rats. Besides, we also found gene *Lpin1* related to triacylglycerol synthesis was significantly upregulated in the exercise GK rats and Wistar rats, which was also found in the human skeletal muscle after exercise (Raue et al., 2012; Vissing and Schjerling, 2014). The mutations in *Lpin1* resulted in systemic insulin resistance, and downregulation of *Lpin1* in C2C12 myotubes via siRNA transfection suppressed insulin action due to the elevated accumulation of intermediate ceramide (Huang et al., 2017). In our data, significantly upregulated *Lpin1* indicated that insulin resistance might be improved in skeletal muscle of exercise GK rats. The gene *Tbc1d1* encodes Tre-2/BUB2/cdc16 domain family member 1 (TBC1D1) which implicated in regulating the traffic of the glucose transporter 4 (GLUT4) and thus modulating glucose uptake (Leto and Saltiel, 2012). Under basal conditions, TBC1D1 prevented GLUT4 translocation by attenuating guanosine triphosphate (GTP) loading of their target Rab GTPases, and silencing of *Tbc1d1* increased the level of surface GLUT4 to promote glucose uptake in the unstimulated L6 muscle cells (Ishikura and Klip, 2008; Cartee and Funai, 2009; Jaldin-Fincati et al., 2017). However, overexpression *Tbc1d1* was required for contraction-induced metabolic responses and deletion of *Tbc1d1* impaired glucose uptake in the exercise-stimulated mice (Stockli et al., 2015; Whitfield et al., 2017; Kjobsted et al., 2019). The significantly upregulated expression of *Tbc1d1* was also observed in the human skeletal muscle after acute exercise (Raue et al., 2012; Vissing and Schjerling, 2014). In the GK rats, *Tbc1d1* expression was increased, indicating that the glucose uptake might be impaired under sedentary condition. Although the gene *Tbc1d1* was further upregulated after exercise in the GK rats, it might be associated with increased contraction-induced glucose uptake. The further study of post-translational modifications of TBC1D1 might help to reveal exercise effects on diabetic GK rats, since it has been reported that phosphorylation of TBC1D1 was increased after exercise and positively associated with contraction-induced muscle glucose uptake, which played an essential role in glucose homeostasis (An et al., 2010; Jessen et al., 2011; Kjobsted et al., 2019).

In the present study, we found a single bout of exercise can improve hyperglycemia in the diabetic GK rats, and RNA-Seq was utilized to investigate the transcriptomic responses of skeletal muscle in GK and Wistar rats with and without exercise. It is the first time to investigate post-acute exercise transcriptomic responses in diabetic GK rats. By integrating our data and previous studies, downregulated *Fasn* and upregulated *Tbc1d1*, *Hk2*, *Lpin1*, *Ppargc1a*, *Sorbs1*, and *Hmox1* might be key regulations to lower plasma glucose and improve insulin sensitivity in the exercise GK rats. Our research provides a new scope for molecular mechanisms on the improvement of hyperglycemia and insulin sensitivity by exercise.

## Ethics Statement

The study was approved by the institutional review board of the Guangdong Key Laboratory of Laboratory Animals. Research protocols conform to the guidelines of the IACUC (Ethics Certificate No.: IACUC2014029).

## Author Contributions

HD and SF conceived and designed the research. SF, YM, and WZ performed the experiments. SF and YM analyzed the data. SF drafted the manuscript. YM, WZ, YH, and HD edited and revised the manuscript. SF, YM, WZ, JW, YH, LH, HC, JK, and HD approved the final version of the manuscript.

## Conflict of Interest Statement

The authors declare that the research was conducted in the absence of any commercial or financial relationships that could be construed as a potential conflict of interest.

## Supplementary Material

FIGURE S1 The levels of plasma glucose and insulin. **(A)** Plasma glucose. **(B)** Plasma insulin. Data were analyzed by two-way ANOVA, and Tukey’s *post hoc* analysis was performed to identify differences between groups. Values are mean ± SEM; *n* = 7–8/group, ^∗∗^*P* < 0.01, ^∗^*P* < 0.05. W, sedentary Wistar group; EW, exercise Wistar group; GK, sedentary GK group; EGK, exercise GK group.

FIGURE S2 The relative expression levels of selected genes measured by qRT-PCR. Data were analyzed by ANOVA, and Tukey’s *post hoc* analysis was performed to identify differences between groups. Values are mean ± SEM; *n* = 4–5/group, ^∗∗^*P* < 0.01, ^∗^*P* < 0.05. Linear regression analysis was performed to determine the correlation coefficient between qRT-PCR and RNA-seq.

FIGURE S3 The highest score modules selected from PPI networks in each comparison. **(A)**–**(C)** represent GK vs. Wistar, exercise Wistar vs. Wistar, and exercise GK vs. GK, respectively. Red nodes indicate upregulated DEGs, and green nodes indicate downregulated DEGs.

TABLE S1 The qRT-PCR primers.

TABLE S2 The details of DEGs among the three comparisons.

TABLE S3 The GO and KEGG pathway enrichment analysis of three comparisons.

TABLE S4 Three clusters of DEGs and related KEGG pathway enrichment analysis.

TABLE S5 The TFs predicted by TRANSFAC.
