# Supplementary figures and images for: Transcriptomic Responses of Skeletal Muscle to Acute Exercise in Diabetic Goto-Kakizaki Rats
Source: Front Physiol. 2019 Jul 9;10:872. doi: 10.3389/fphys.2019.00872 (PMC6629899; doi:10.3389/fphys.2019.00872)

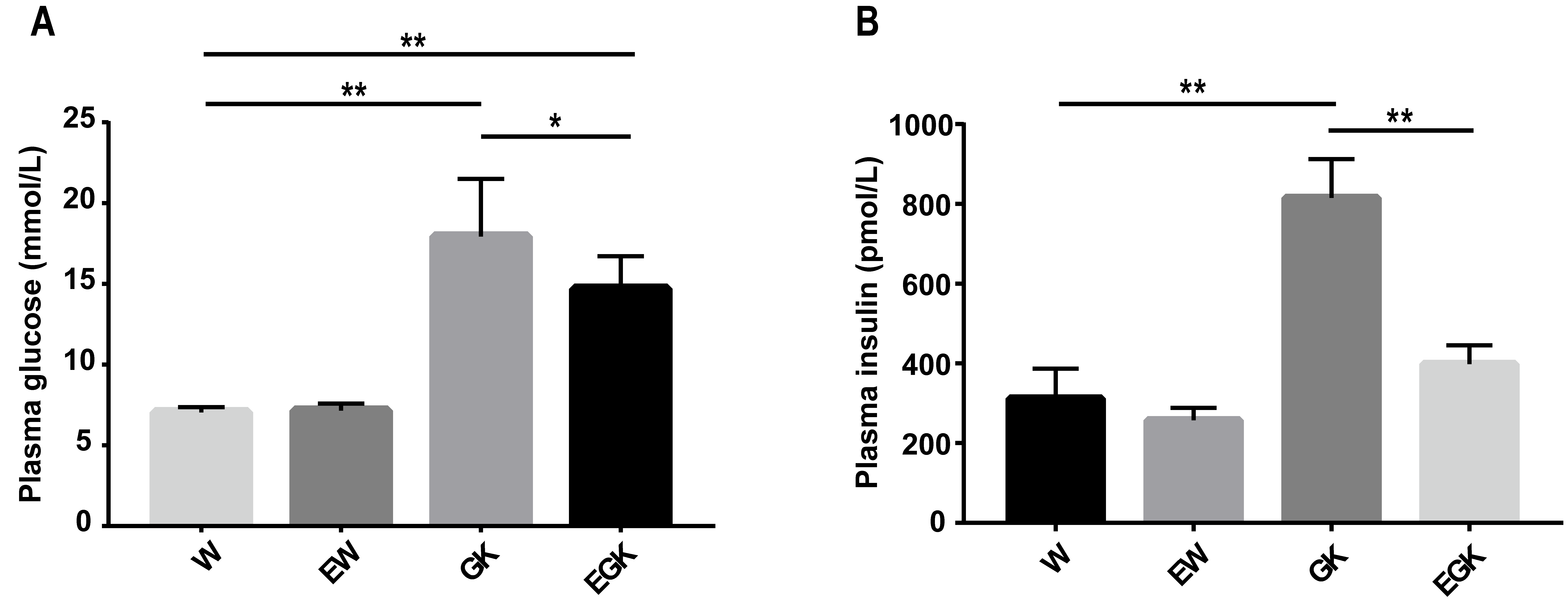

Supplement: FIGURE S1 — The levels of plasma glucose and insulin. (A) Plasma glucose. (B) Plasma insulin. Data were analyzed by two-way ANOVA, and Tukey’s post hoc analysis was performed to identify differences between groups. Values are mean ± SEM; n = 7–8/group, ∗∗P < 0.01, ∗P < 0.05. W, sedentary Wistar group; EW, exercise Wistar group; GK, sedentary GK group; EGK, exercise GK group. [file Image_1.TIF]

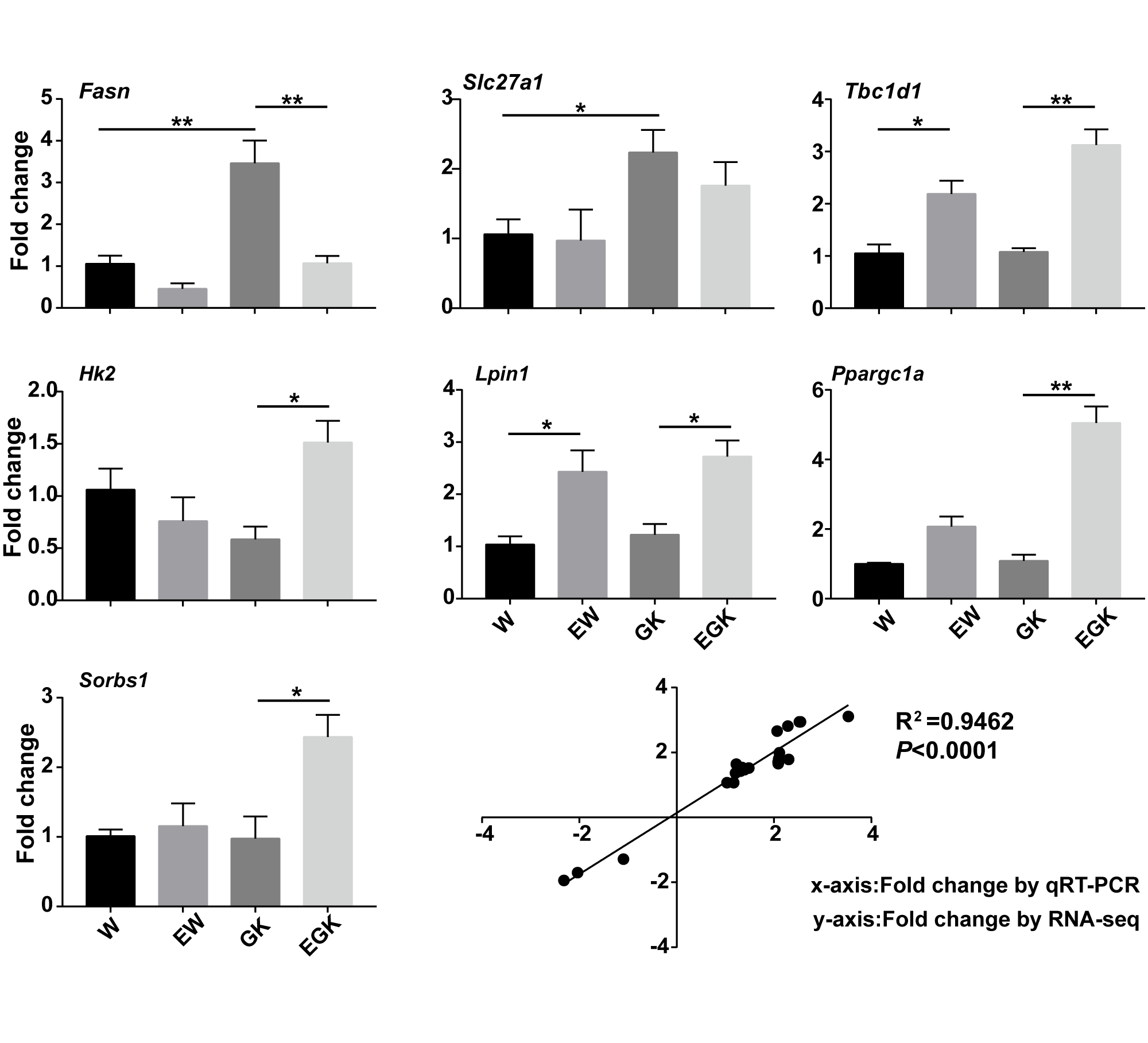

Supplement: FIGURE S2 — The relative expression levels of selected genes measured by qRT-PCR. Data were analyzed by ANOVA, and Tukey’s post hoc analysis was performed to identify differences between groups. Values are mean ± SEM; n = 4–5/group, ∗∗P < 0.01, ∗P < 0.05. Linear regression analysis was performed to determine the correlation coefficient between qRT-PCR and RNA-seq. [file Image_2.TIF]

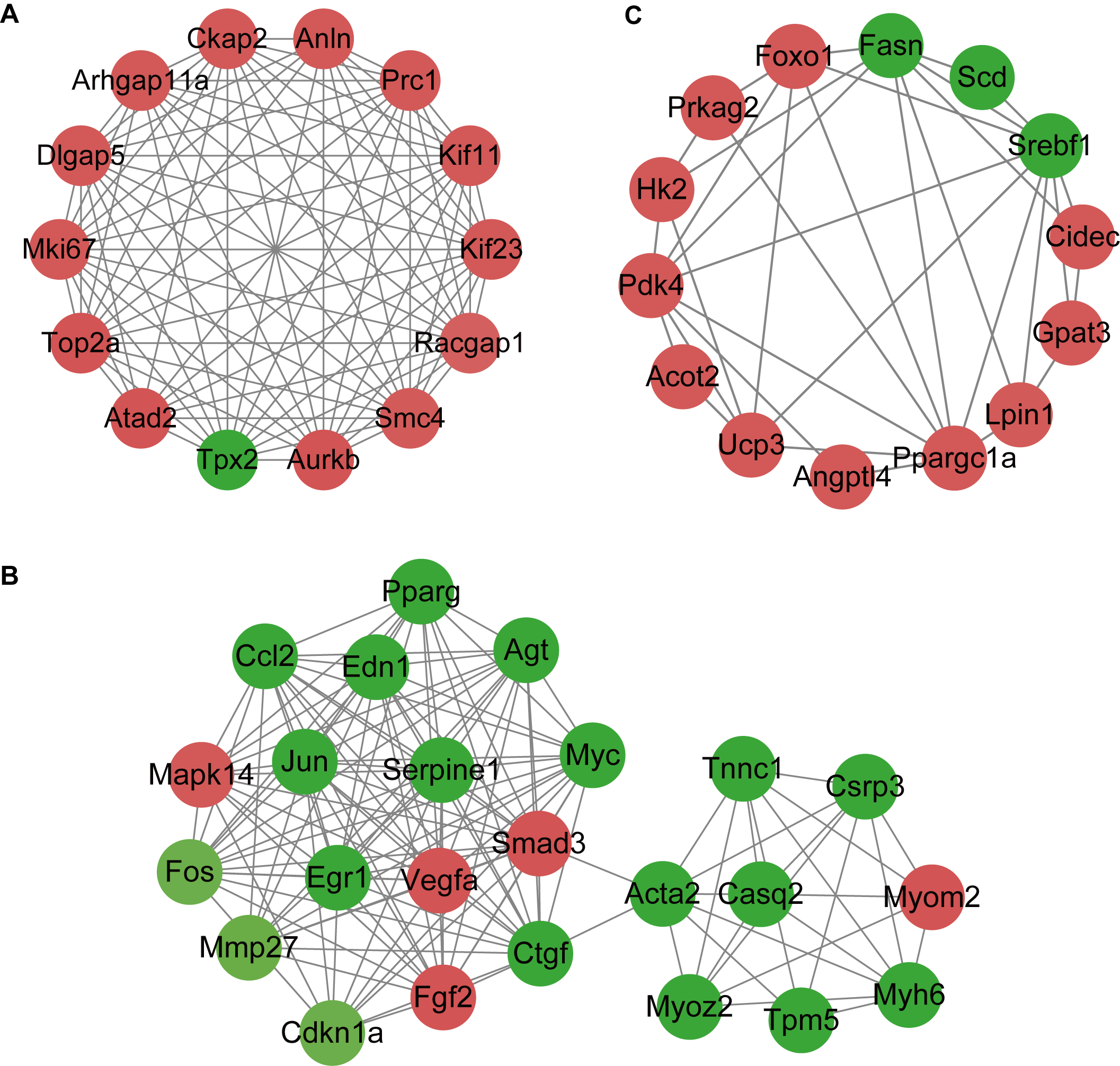

Supplement: FIGURE S3 — The highest score modules selected from PPI networks in each comparison. (A)–(C) represent GK vs. Wistar, exercise Wistar vs. Wistar, and exercise GK vs. GK, respectively. Red nodes indicate upregulated DEGs, and green nodes indicate downregulated DEGs. [file Image_3.TIF]
